# Anti-Carbamylated Protein (Anti-CarP) Antibodies in Patients Evaluated for Suspected Rheumatoid Arthritis

**DOI:** 10.3390/diagnostics12071661

**Published:** 2022-07-08

**Authors:** Vincent Ricchiuti, Kelly Y. Chun, Jane M. Yang, Mary Ann Aure, Luis Gomez, Gary L. Norman, Michael Mahler

**Affiliations:** 1 LabCorp North Central Regional Reference Laboratory, Dublin, OH 43016, USA; ricchiv@labcorp.com; 2 LabCorp Esoterix, Calabasas, CA 91301, USA; chunk@labcorp.com (K.Y.C.); yangj5@labcorp.com (J.M.Y.); 3 Headquarters & Technology Center Autoimmunity, Werfen, San Diego, CA 92131, USA; maure@werfen.com (M.A.A.); lgomez1@werfen.com (L.G.); gnorman@werfen.com (G.L.N.)

**Keywords:** anti-carbamylated protein, rheumatoid arthritis, prognosis, rheumatoid factor (RF), 14-3-3 eta, anti-citrullinated protein antibodies (ACPA), cyclic citrullinated peptide (CCP)

## Abstract

(1) Background: Anti-carbamylated protein (CarP) antibodies have been studied as novel markers to aid in the diagnosis and prognosis of rheumatoid arthritis. (2) Methods: A total of 265 samples were included in the evaluation, for which 98 had results for anti-cyclic citrullinated peptide (CCP), 86 for rheumatoid factor (RF), and 212 for 14-3-3 eta protein. Anti-CarP antibodies were measured using a fetal calf serum-based single-step assay (research use only, Inova Diagnostics, San Diego, CA). (3) Results: Anti-CarP antibodies were significantly higher and more frequent in anti-CCP3.1+ (*p* = 0.0025), RF+ (*p* = 0.0043) and 14-3-3 eta+ (*p* = 0.028) samples compared to the negative counterpart group. In addition, isolated anti-CarP positivity occurred in samples negative for anti-CCP3.1, RF, or 14-3-3 eta. When anti-CarP antibodies were compared to each of the RF, anti-CCP3.1, and 14-3-3 eta by receiver operating characteristic (ROC) analyses, the area under the curve (AUC) values of 0.71 (RF), 0.68 (anti-CCP3.1), and 0.59 (14-3-3 eta), respectively, demonstrated a moderate correlation. Using an UpSet plot, we determined that 10.6% of the samples with available results for anti-CCP3.1, RF, and anti-CarP showed triple positivity. (4) Conclusions: Anti-carbamylated protein (anti-CarP) antibodies can be detected in anti-CCP, RF and 14-3-3 eta-positive and -negative patients, potentially identifying specific subsets of patients.

## 1. Introduction

With a prevalence of approximately 1%, rheumatoid arthritis (RA) is one of the most common autoimmune disorders worldwide [1]. Treatment at early stages can significantly slow disease progression and potentially prevent irreversible damage. However, early diagnosis is often challenging due to the lack of serological markers. Up to 50% of early RA patients are negative for the standard serological markers rheumatoid factor (RF) or anti-citrullinated protein antibodies (ACPA) including anti-cyclic citrullinated peptide (CCP). While many studies have aimed to identify novel biomarkers with the potential to close the serological gap in RA, many patients remain unrecognized, and early treatment initiation is delayed.

**Figure 1:**
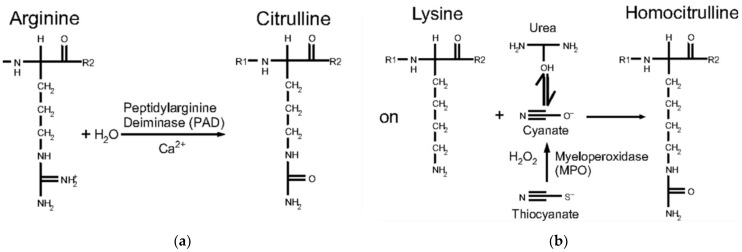
Citrullination (**a**) and carbamylation (**b**) occurring on different amino acids via different mechanisms but yielding similar end-products (Adapted from Shi et al., 2011 [2]).

In 2011, antibodies directed against carbamylated protein antigen (anti-CarP antibodies) were identified in RA patients, and subsequent studies have established the predictive and prognostic value of this antibody system [2,3]. Unlike citrullination, which is an enzyme-mediated modification of arginine to citrulline, carbamylation occurs through the chemical modification of lysine residues with cyanide to form homo-citrulline. Homo-citrulline is very similar to citrulline, with the only difference being the addition of one CH2 to its side chain (Figure 1).

In addition, evidence is mounting in support of the value of combined testing for anti-CCP3.1, 14-3-3 eta, RF, and anti-CarP antibodies, and especially for triple positivity (anti-CCP, RF and anti-CarP), conferring a very high likelihood ratio for RA [2,4,5,6,7,8,9,10,11]. Most previous reports describing anti-CarP assay performance characteristics have been derived from European populations using a two-step research assay [2,7] in which specimens need to be tested on one microwell plate coated with carbamylated fetal calf serum (FCS) and one with uncarbamylated FCS [2,3,7]. The final value is obtained by subtracting the value obtained on the uncarbamylated FSC from that obtained on the carbamylated plate.

In the present study, we assess anti-CarP measurements using a novel single-step assay that obviates the two-step process, saving time and labor and minimizing specimen handling, and is thus highly preferable for a large clinical reference laboratory [11]. Our dual objectives in this current study were to assess the performance of the single-step anti-CarP assay and to compare anti-CarP to established RA markers in a cohort of real-life patient samples submitted to a United States clinical reference laboratory for RA diagnostic work-up.

## 2. Materials and Methods

### 2.1. Patient Characteristics

From April to November 2021, a total of 265 samples were selected from clinical specimens submitted for routine RA testing based on RF, anti-CCP, and/or 14-3-3 eta positivity. Of 265, 98 had results for anti-CCP3.1 IgG/IgA, 86 for RF, and 212 for 14-3-3 eta. A total of 65 samples had results for all four analytes. All of the patients were adults (>18 years old).

### 2.2. Immunoassays

Anti-CarP antibodies were measured using a fetal calf serum-based single-step assay (research use only, Inova Diagnostics, San Diego, CA, USA), as previously described [12]. Of the 265 patients, 98 had also results for anti-CCP3.1, 86 for rheumatoid factor (RF), and 212 for 14-3-3 eta.

Anti-cyclic citrullinated peptide (CCP) was measured using CCP3.1, Inova Diagnostics, San Diego, CA; rheumatoid factor (RF), Roche Diagnostics Corporation, Indianapolis, IN; and 14-3-3 eta protein, AugureX, Vancouver, BC, Canada.

### 2.3. Statistical Analysis

Receiver operating characteristic (ROC) analysis was used to analyze the discriminatory ability of the different antibodies (Using DataLab, Werfen). The Wilcoxon–Mann–Whitney test was used for pairwise comparisons. Spearman correlation analysis was performed to evaluate the association between the different biomarkers. *p*-values less than 0.05 were considered statistically significant. Visualization of the overlap in positivity for various biomarker combinations was accomplished using ‘UpSet plots’ utilizing software library libraries in Python, as described by Lex and colleagues. (Python, version 3.8; Matplotlib, version 3.3.2; UpSetPlot, version 0.4.1). Correlation coefficients were used according to Mukaka et al. (2012) [13].

## 3. Results

**Figure 2:**
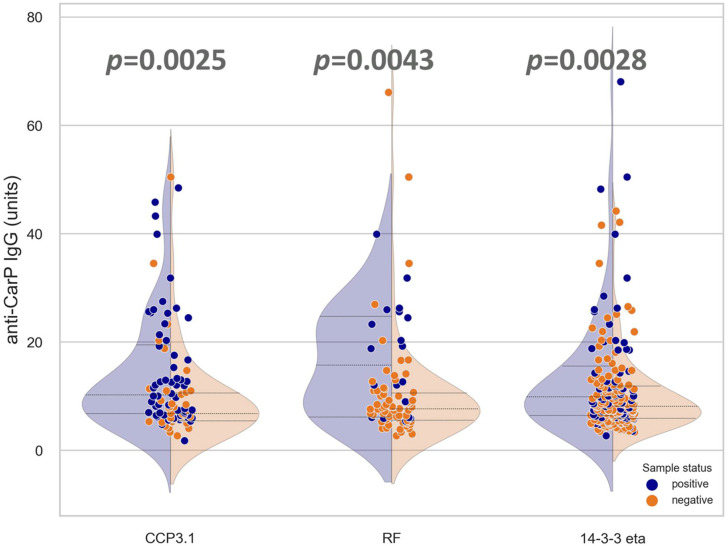
Combined violin and dot plot showing the anti-CarP autoantibody levels among patients characterized as positive/negative for the markers anti-CCP3.1, RF, and 14-3-3 eta. Blue and orange dots represent positive and negative results, respectively, per assay outlined in the *x*-axis. *p*-values indicate significant differences between anti-CarP IgG median unit values for comparator groups.

Anti-CarP antibodies were significantly higher and more frequent in anti-CCP3.1+ (*p* = 0.0025), RF+ (*p* = 0.0043), and 14-3-3 eta+ (*p* = 0.028) samples compared to the negative counterpart group (Figure 2).

### 3.1. Anti-CarP Antibodies Are Found in Both ACPA-Positive and ACPA-Negative Patient Samples

In addition, isolated anti-CarP positivity occurred in samples negative for anti-CCP3.1, RF, and 14-3-3 eta. When the anti-CarP antibodies were compared to anti-CCP3.1, RF, and 14-3-3 eta by receiver operating characteristic (ROC) analysis with the three markers as a binary reference, area under the curve (AUC) values of 0.68 (anti-CCP3.1), 0.71 (RF), and 0.59 (14-3-3 eta) were observed.

### 3.2. Association and Correlation of Anti-CarP Antibodies with Anti-CCP3.1, RF, or 14-3-3 Eta

**Figure 3:**
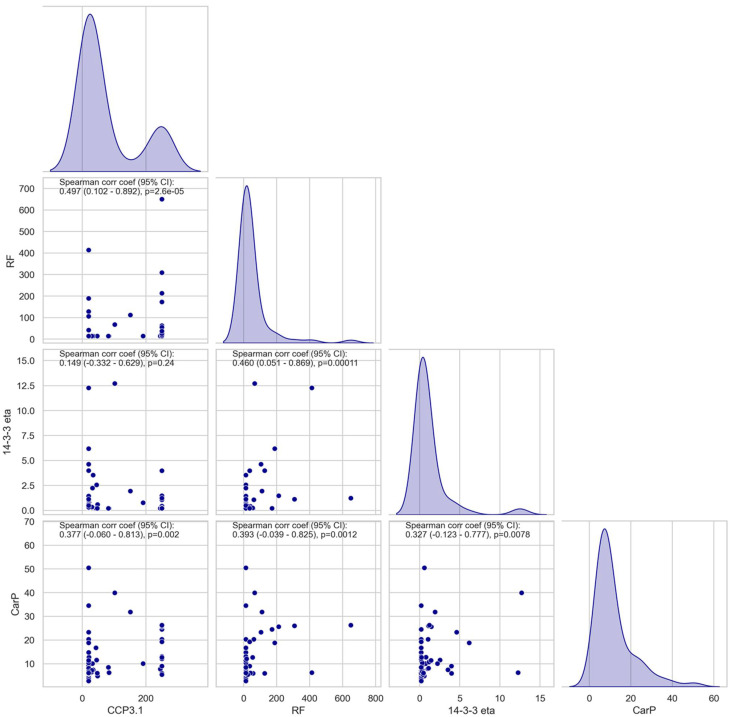
Correlation diagram between rheumatoid arthritis (RA)-associated markers anti-CarP, anti-CCP3.1, RF, and 14-3-3 eta. Spearman’s coefficients and *p*-values are indicated in the figure.

**Figure 4:**
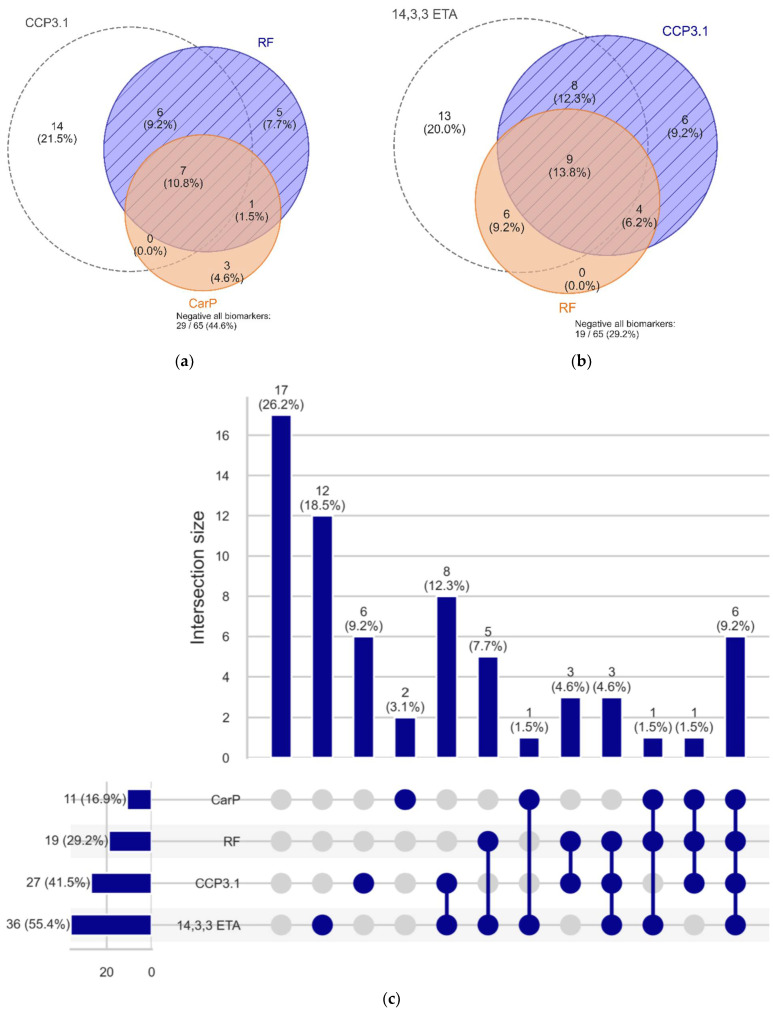
Overlap between anti-CCP3.1, RF, 14-3-3 eta, and anti-CarP antibodies. Panel (**a**) shows a Venn diagram of anti-CCP3.1, RF, and anti-CarP antibodies. Panel (**b**) shows a Venn diagram of anti-CCP3.1, RF, and 14-3-3 eta. Panel (**c**) illustrates the combination of the four markers using an UpSet plot.

A moderate quantitative correlation was observed between anti-CarP and other markers. The highest agreement was found with RF (rho = 0.393) followed by anti-CCP3.1 (0.377) and 14-3-3 eta (0.327) (Figure 3). The correlations were lower than anti-CCP3.1 vs. RF (0.497), but significantly higher than anti-CCP3.1 and 14-3-3 eta (0.149). Using an UpSet plot, we determined that 9.2% of the samples with available results for anti-CCP3.1, RF, anti-CarP, and 14-3-3 eta showed quadruple positivity. (Figure 4).

## 4. Discussion

In both JIA and adult forms of RA, the disease may progress for variable time periods before symptoms emerge. Although patients may be asymptomatic, it has been recognized for many years that the levels of various biomarkers such as RF, C- reactive protein (CRP), and anti-CCP increase prior to the appearance of symptoms [14,15]. The early detection of developing RA offers the potential to institute therapeutic treatment early in the disease course and slow or prevent irreversible joint damage [16,17]. However, despite the good performance of anti-CCP antibodies, additional markers are needed to detect the estimated 30-40% of patients who are seronegative for anti-CCP and RF, especially in the critical early stages when intervention may be the most effective [16,17,18]).

Among the novel RA markers, anti-CarP antibodies have been described in both APCA-positive and ACPA-negative RA patients [2,3,19]. A recent study reported that 23.6% of RA patients negative for ACPA and RF were anti-CarP-positive [3]. Importantly, these antibodies can also be detected prior to symptom onset [2,7,10,18,20].

In addition to providing increased diagnostic value, anti-CarP antibodies have been reported to correlate with radiological progression independent of ACPA and to thus provide important prognostic insight for more effective management [3,19,21]. Furthermore, anti-CarP antibodies have been associated with increased mortality in patients with RA [22]. The original method described by Shi et al. for the detection of anti-CarP antibodies involved the testing of antibodies with carbamylated and non-carbamylated fetal calf serum (FCS) by ELISA [2]. Subsequently, additional tests using a wide range of carbamylated antigens have been described, and studies have confirmed the heterogeneity of anti-CarP antibodies in patients [23,24,25,26]. Although some novel carbamylated autoantigens hold promise; presently, the observed heterogeneity in anti-carbamylated antibodies suggests that FCS might offer the best way to detect the broad polyclonal anti-CarP B-cell response expressed by RA patients. Consequently, the ELISA used in the present study follows a protocol using FCS, with the single modification being that only carbamylated FCS is used [12].

The diagnostic value of anti-CarP has been a matter of discussion. A meta-analysis performed in 2016 by Li et al. revealed a sensitivity and specificity of 42% and 96%, respectively [27]. A recent study reported a similar overall sensitivity of 46.7% and detection of 4.1% in RA patients who were ACPA- and RF-negative [28].

The data presented in this study provide further evidence that anti-CarP antibodies can be found in ACPA- and/or RF-negative patients. Although we do not have a clinical diagnosis for the individuals in our referral cohort, we found that 4.5% of the ACPA- and RF-negative specimens were positive for anti-CarP antibodies. 14-3-3-eta is another marker with potential value to characterize patients with RA and as a supplementary marker for RF and CCP [29]. Our study is one of the few investigations analyzing anti-CarP antibodies in parallel to 14-3-3 eta. We found anti-CarP and 14-3-3 eta antibodies complemented the other three markers. Anti-CarP antibodies and anti-14-3-3 eta exclusively detected 3.1 and 18.5% of the tested specimens, respectively. Note that the specificity of the markers was not assessed in this evaluation. The correlation between anti-CarP, ACPA, and RF is in line with previous studies. In addition, we found similar correlations between anti-CarP and 14-3-3 eta, as described by Zhang et al. [30].

The determination of the value of new biomarkers requires the testing of multiple markers on the same cohort to assess additive values as well as overlapping reactivities, as shown in Figure 4. This can be especially difficult for assays that are still in a research phase and that are not readily available. Unless biomarkers are tested on the same cohort, however, it is not possible to accurately compare the effectiveness of different combinations of biomarkers. While multiple biomarkers may be increased in a particular disease, it is critical that each marker adds value and does not provide redundant information that does not improve patient diagnosis or management. The one situation in which redundant positivity may be of value, however, is when the presence of multiple biomarkers has proven clinical significance. This was shown in a recent meta-analysis, where triple positivity (ACPA + RF + anti-CarP) was strongly associated with early RA [26].

An intrinsic limitation of studies conducted at large reference laboratories using routine samples is the availability of clinical data. However, comparing the results for anti-CarP antibodies with markers used in routine evaluation provides valuable insights into the prevalence, overlap, and potential value of the markers. As expected, we found overlap between anti-CarP, anti-CCP3.1, and RF, but also found samples with reactivity to anti-CarP only.

## 5. Conclusions

Our data confirm the association of anti-CarP antibodies with anti-CCP3 and RF and expand the knowledge with data on the association with 14-3-3 eta. The moderate overlap between the three main markers (anti-CCP3, RF, and anti-CarP) might indicate synergistic characteristics. As the field increasingly looks for precision medicine models for diagnosis and patient management, the identification, validation, and availability of well-characterized biomarkers is an important element for improving patient care and outcomes.
